# Identifying obstacles hindering the conduct of academic-sponsored trials for drug repurposing on rare-diseases: an analysis of six use cases

**DOI:** 10.1186/s13063-022-06713-y

**Published:** 2022-09-15

**Authors:** Marta del Álamo, Christoph Bührer, Dirk Fisher, Matthias Griese, Paul Lingor, Giovanni Palladini, Nicolas Sireau, Virginie Hivert, Luca Sangiorgi, Florence Guillot, Juliane Halftermeyer, Lenka Soucková, Kristýna Nosková, Regina Demlová

**Affiliations:** 1 ECRIN, European Clinical Research Network Infrastructure, Paris, France; 2 Department of Neonatology, Charité Universitätsmedizin, Berlin, Germany; 3 Division of Developmental- and Neuropaediatrics, University Children’s. Hospital Basel (UKBB), University of Basel, Spitalstrasse 33, Postfach, 4031 Basel, Switzerland; 4 Department of Pediatrics, Dr. von Hauner Children’s Hospital, University. Hospital, LMU Munich, German Center for Lung Research (DZL), Lindwurmstraße 4, 80337 Munich, Germany; 5 Department of Neurology, Klinikum rechts der Isar, Technical University of Munich, Munich, Germany; 6 Amyloidosis Research and Treatment Center, Foundation “Istituto di Ricovero e Cura a Carattere Scientifico (IRCCS) Policlinico San Matteo,” and Department of Molecular Medicine, University of Pavia, Pavia, Italy; 7 AKU Society, Cambridge, UK; 8 EURORDIS- Rare Diseases Europe, Paris, France; 9 Department of Medical Genetics and Skeletal Rare Diseases, IRCCS Rizzoli Orthopaedic Institute, Bologna, Italy; 10 ANR, French National Research Agency, Paris, France; 11 Inserm Transfert, Paris, France; 12 Department of Pharmacology, Faculty of Medicine, Masaryk University, Brno, Czech Republic

**Keywords:** Randomized clinical trials, Rare diseases, Drug repurposing, Academic-sponsored, Barriers, Challenges

## Abstract

**Background:**

Academic-sponsored trials for rare diseases face many challenges; the present paper identifies hurdles in the set-up of six multinational clinical trials for drug repurposing, as use cases.

**Methods:**

Six academic-sponsored multinational trials aiming to generate knowledge on rare diseases drug repurposing were used as examples to identify problems in their set-up. Coordinating investigators leading these trials provided feedback on hurdles linked to study, country, and site set up, on the basis of pre-identified categories established through the analysis of previous peer-reviewed publications.

**Results:**

Administrative burden and lack of harmonization for trial-site agreements were deemed as a major hurdle. Other main identified obstacles included the following: (1) complexity and restriction on the use of public funding, especially in a multinational set up, (2) drug supply, including procurement tendering rules and country-specific requirements for drug stability, and (3) lack of harmonization on regulatory requirements to get trial approvals.

**Conclusion:**

A better knowledge of the non-commercial clinical research landscape and its challenges and requirements is needed to make drugs—especially those with less commercial gain—accessible to rare diseases patients. Better information about existing resources like research infrastructures, clinical research programs, and counseling mechanisms is needed to support and guide clinicians through the many challenges associated to the set-up of academic-sponsored multinational trials.

## Background

Many barriers exist to advancing knowledge and treatment for rare diseases. The most acknowledged challenge is the small number of eligible participants for a given study, with a major impact in the design and coordination of randomized clinical trials (RCTs), which are still the basis for the evaluation of interventions and clinical guidelines. In this set up, multinational, multi-center clinical trials are required to achieve sufficient recruitment. International collaboration is nevertheless constrained by scientific, ethical, economic and regulatory considerations.

In 2008, Duley and collaborators [1] identified major barriers to conduct of RCTs for all diseases areas through a systematic literature review. More recently similar analysis has been published for all disease areas [2], rare diseases [3], and developing countries [4]. Although rare diseases may present unique clinical problems, some of the methodological and operational challenges to studying health outcomes are common for other diseases areas. Indeed, all these publications have similarly concluded that the barriers to the conduct of multinational RCTs are significant, and although none of these reviews make a clear distinction between industry-sponsored trials and academic/investigator-initiated trials, most of the identified constraints might affect especially the latter [5].

Commercially sponsored clinical trials are responsible for bringing most of the new drugs to the market. However, these clinical trials assess the safety and efficacy of drugs that are chosen by a commercial entity that funds the entire process. Non-commercial or academic trials have their own additional specific objectives, often focusing on refining or getting new indications of available treatments (drug repurposing) and optimizing therapeutic strategies that do not have as much financial gain for the pharmaceutical industry. Drug repurposing has especially been coined as a possible relevant strategy for development of medicines for rare diseases. Indeed, a recent analysis [6] showed that 75% of the developments of repurposed orphan drugs authorized during the 2016–2020 period were started in academia. Pharmaceutical companies may find a commercial interest in pursuing clinical development to repurpose drugs that are still on patent. However, they are less likely to carry out such a development for out-of-patent medicines. Academic institutions would be willing to explore repurposing options despite the fact that academic-sponsored trials face a high number of challenges, including (a) lack of funding or long ranging financial support covering unexpected issues during the trial (b) inadequate infrastructure to plan, implement, and execute the trial including a working quality management system (c) necessary structured processes to facilitate academic collaborations, and a lack of platforms to discuss and solve issues related to academic trials that results in difficulties to access the right partner assisting on the operational management, usually provided by contract research organizations (CROs) on industry-sponsored trials.

Academic sponsors and investigators end-up getting involved not only in the scientific aspects of the research, but also having to navigate the operational coordination and management themselves.

Because drug repurposing has the potential to deliver treatments faster to patients with rare diseases, in this study, we address the following research question—what are the hurdles than hinder the conduct of clinical trials on drug repurposing for rare diseases initiated by academia? This paper describes outcomes of a use cases analysis of the methodological and operational challenges faced by coordinating investigators leading 6 investigator-initiated trials for drug repurposing on rare diseases over the last five years, most of them funded through a “virtual common pot” mechanism, i.e., through the combination of national funding sources, without cross-border transfer of funds.

## Methods

This work is based on topics discussed in two meetings, the mid-term monitoring meeting of E-Rare3 JTC2016 (organized in 2020) and the European Joint Program Networking Support Scheme (EJP NSS) workshop “Identifying systematic obstacles hindering the development of academic-sponsored trials for drug repurposing on rare-diseases.” The E-rare3 2020 mid-term Monitoring Meeting followed the development and advances of the projects granted on the E-Rare3-Joint Translational Call 2016 (“Clinical research for new therapeutic uses of already existing molecules-repurposing-in Rare Diseases”). This was the first time that a Rare Diseases Joint Transnational Call funded clinical trials and 8 projects were selected. Five of the 8 investigators awarded on this call, who presented the progress of their funded trials in 2020, were later invited to participate in the EJP NSS workshop, aiming to discuss and identify operational hurdles linked to the development of multinational academic-sponsored trials for rare diseases. Additionally, the NSS event included the communication of the DevelopAKUre drug development program. This program was not funded through the E-Rare3-JTC2016 but comprised three multinational academic-sponsored trials (SONIA1, SONIA2 and SOFIA) for drug repurposing partly funded by the EU (FP7 program) and was considered of high interest to reach the goals of the NSS event. Thus, six academic-sponsored multinational RCTs for rare diseases were selected as use cases to describe operational hurdles (Table 1). The 3 trials integrating the DevelopAKUre program were considered all together as one “use-case,” provided that they were all coordinated by the same team and had a similar operational set up.

**Table 1:** Summary of the 6 academic-sponsored multinational RCTs for rare diseases selected as use cases to describe operational hurdles

Project	Trial/ClinicalTrials.gov (NCT)	Protocol/results
**HCQ4SurfDefect**	Hydroxychloroquine (HCQ on Paediatric Interstitial Lung Disease (ILD) (HCQ-chILD-EU) NCT02615938	Prospective evaluation of hydroxychloroquine in pediatric interstitial lung diseases: Study protocol for an investigator-initiated, randomized controlled, parallel-group clinical trial [7] Randomized controlled phase 2 trial of hydroxychloroquine in childhood interstitial lung disease [8]
**Redox**	A trial of Doxycycline vs. Standard Supportive Therapy in Newly-diagnosed Cardiac AL Amyloidosis Patients Undergoing Bortezomib-based Therapy NCT03474458	Protocol not published
**ROPROP**	Oral propranolol for prevention of threshold retinopathy of prematurity (ROPROP) NCT03083431	Oral propranolol for prevention of threshold retinopathy of prematurity (ROPROP): protocol of a randomized controlled trial [9]
**ROCK ALS**	Inhibition of Rho Kinase (ROCK) with fasudil as disease-modifying treatment for ALS (ROCK-ALS) NCT03792490	ROCK-ALS: Protocol for a Randomized, placebo-controlled, double-blind Phase IIa trial of safety, tolerability and efficacy of the Rho kinase (ROCK) inhibitor fasudil in Amyotrophic Lateral Sclerosis [10]
**TAM DMD**	Tamoxifen in Duchenne Muscular Dystrophy (TAMDMD) NCT03354039	Tamoxifen in Duchenne muscular dystrophy (TAMDMD): study protocol for a multicenter, randomized, placebo-controlled, double-blind phase 3 trial [11]
**DevelopAKUre Program** **SONIA 1**	Dose response study of nitisinone in Alkaptonuria (SONIA 1) NCT01828463	Suitability of nitisinone in alkaptonuria 1 (SONIA 1): an international, multicenter, randomized, open-label, no-treatment controlled, parallel-group, dose-response study to investigate the effect of once daily nitisinone on 24-h urinary homogentisic acid excretion in patients with alkaptonuria after 4 weeks of treatment [12]
**DevelopAKUre Program** **SONIA2**	Suitability of nitisinone in alkaptonuria 2 (SONIA 2) NCT01916382	Efficacy and safety of once-daily nitisinone for patients with alkaptonuria (SONIA 2): an international, multicenter, open-label, randomized controlled trial [13]
**DevelopAKUre Program** **SOFIA**	SOFIA	Subclinical ochronosis features in Alkaptonuria: A cross-sectional study [14]

All 6 use cases were selected based on the following criteria:

- Multinational clinical trial for rare diseases
- Investigator-initiated trial
- Drug repurposing

and availability of their coordinators to participate in the EJP NSS workshop.

Coordinating investigators were asked to provide feedback regarding main obstacles hindering the development of the trials according to the following categories and sub-categories:

1. Study set up

- Study design
- Funding and funding mechanism
- Sponsorship
- Drug procurement
- Project management
- Data management
- Vigilance
- Monitoring

2) Country set up

- Country selection
- National Competent Authority (NCA)/Ethics Committee (EC) approval
- Insurance
- GCP requirements

3) Site set up

- Site agreements
- Site compliance

This categories’ classification is based on main pre-identified barriers described in previous publications:

- Articles describing systematic barriers, opportunities, and lessons learned in the set-up of academic-sponsored international multi-center clinical trials for rare diseases [15–17]
- Systematic reviews aiming to identify barriers to the conduct of RCT (Table 2)

**Table 2:** Major barriers to the conduct of randomized trials identified by systematic reviews

**Major barriers to the conduct of randomized trials**	**Source**
Inadequate funding	Duley et al. (2008) [1], Djurisic et al. (2017) [2], Alemayehu et al. (2018) [4]
Complex/not harmonized regulations	Duley et al. (2008) [1], Djurisic et al. (2017) [2], Alemayehu et al. (2018) [4]
Excessive/non-focused monitoring	Duley et al. (2008) [1], Djurisic et al. (2017) [2]
Over-restrictive interpretations of privacy laws without evidence of subject benefit/Lack of transparency	Duley et al. (2008) [1], Djurisic et al. (2017) [2]
Inadequate understanding of methodology	Duley et al. (2008) [1]
Inadequate identification of the clinical research questions	Djurisic et al. (2017) [2]
Inadequate knowledge and understanding of clinical research	Djurisic et al. (2017) [2]
Inadequate knowledge and understanding of clinical trials	Djurisic et al. (2017) [2]
Inadequate infrastructures	Djurisic et al. (2017) [2], Alemayehu et al. (2018) [4]
Unsupportive administrative system	Alemayehu et al. (2018) [4]
Competing demands	Alemayehu et al. (2018) [4]
Difficult patient recruitment	Alemayehu et al. (2018) [4]
**Major barriers to the conduct of RCTs in rare diseases**	**Source**
Difficult to recruit patients due to rarity	Rath et al. (2017) [3]
Incomplete understanding of national history to inform trial design	Rath et al. (2017) [3]
Need for trial designs adapted to small population size and clinical heterogeneity	Rath et al. (2017) [3]
Organizational challenges as a consequence from the need for multinational randomized clinical trials	Rath et al. (2017) [3]
Need for more sensitive outcome measures to quantify disease	Rath et al. (2017) [3]
Need for involvement of all the stakeholders in the study design and conduct	Rath et al. (2017) [3]

Barriers identified by these 7 sources were all compiled. All listed barriers were asked to be considered for the “use cases” analysis.

Data were collected from investigator’s presentations during the NSS meeting and the discussions held after the “use cases” presentations. Presentations were structured as per the listed categories. Information was structured as shown in Table 3, which was fully reviewed by all investigators during the manuscript’s preparation, to ensure accuracy.

**Table 3:** Major barriers to the conduct of randomized trials for rare diseases identified by use cases on RCT for drug repurposing

STUDY SET UP	Comments/rational	Identified as a hurdle (trial) (number of trials)
**Study design**	Lack of natural history studies to inform about trial design Lack of registries validated surrogate outcomes and patient reported outcome measures to choose an appropriated study design Small and heterogeneous patients groups (risk of being underpowered to test efficacy)	HCQ4SurfDefect (1/6)
**Funding and funding mechanism**	Non-commercial sponsors often face budgetary problems due to lack of public funding opportunities and/or poor flexibility of the external funding	ROP ROP, Redox, DevelopAKUre, ROCK-ALS, TAM DMD, HCQ4SurfDefect (6/6)
**Sponsorship**	Lack of experience and/or insufficient knowledge of academic organizations about sponsor responsibilities in a multinational setting Lack of trial-specific legal support Co-sponsorship not yet possible in Europe	Redox, ROCK-ALS, HCQ4SurfDefect (3/6)
**Drug procurement**	Tendering process to select drug supplier Custom restrictions Country-specify requirements for data on drug stability Lack of placebo suppliers	Redox, ROCK-ALS, HCQ4SurfDefect (3/6)
**Project management**	Lack of experience in multinational trials in legal affairs department	ROP ROP (1/6)
**Data management**	Lack of harmonization among countries Lack of experience on multinational trials from selected data centers	Not reported
**Vigilance (PV)**	Lack of harmonization among countries Lack of experience on multinational trials from selected PV center	Redox (1/6)
**Monitoring**	Lack of harmonization on monitoring plan requirements	Not reported
**COUNTRY SET UP**		
**Country selection**	Scarcity of patients leads to large-scale studies, i.e., multicenter/multinational set-ups	Not reported
**NCA/EC approval/regulatory**	Timing/unexpected delays Lack of harmonization	ROP ROP, ROCK-ALS, TAM DMD (3/6)
**Insurance**	Lack of harmonization on requirements, minimum coverage varies among EU countries	ROCK-ALS (1/6)
**Good Clinical Practice (GCP) requirements**	Lack of harmonization on GCP training content/extent	ROP ROP (1/6)
**SITE SET UP**		
**Site agreements**	Lack of harmonization on site agreements templates even within the same country Country-specific terminology/interpretation (legal responsibilities, intellectual/industrial property, GDPR) included in site contracts, reference to the national, legal and regulatory framework (liability and insurance), templates tailored to industry sponsored trials, need to align with Consortium/Grant Agreements	ROP ROP, Redox, TAM DMD, HCQ4SurfDefect (4/6)
**Site compliance**	Lack of personnel experienced in clinical research Lack of personnel involved in academic trials Personnel’s turnover due to trial’s extensions	Not reported

Investigators were also asked to provide feedback regarding any possible additional barrier not previously identified.

## Results and discussion

Table 3 summarizes the main findings on the analysis of the 6 RCTs presented and discussed during the 2020 E-Rare3 and EJP RD NSS meetings.

### Study set up

#### Study design

Lack of natural history studies to inform about trial design, lack of registries and patient reported outcome measures are some of the possible obstacles to choose an appropriate study design [3, 17]. Patient groups are very small and heterogeneous and the risk of being underpowered to test efficacy is high. The need to develop cost-efficient and novel trial designs and analysis to study efficacy in heterogeneous, small populations has led to the development of specific research programs funded by the European Commission to work on these topics [18–20], and the International Rare Diseases Research Consortium (IRDiRC) has published recommendations for the design of small population clinical trials [21]. Nevertheless, implementation of novel methodologies might still be suffering of “methodology aversion” [22] that jeopardizes the regulators’ acceptance of useful methods that might not be fully understood by stakeholders not involved in its development. As an attempt to improve the dialogue between academia and regulators the STARS project [23] aims to reach out to medicine innovators in academia to bridge the regulatory knowledge gap which might prevent the access of patients to these innovations.

Only one of the coordinating investigators of the RCTs analyzed in this work reported the study design as an obstacle. This perception might reflect that this challenge prevents investigators to opt for innovative trial designs and/or embark on setting up the trial, even from applying for funding. Thus, this topic might have been disregarded as a constraint by the investigators involved in these studies. It is worth noting that two of the studies (DevelopAKUre program studies SONIA 1, SONIA 2 and SOFIA and TAM DMD trials) looked for scientific advice (EMA and/or National Competent Authority), taking advantage of the several opportunities opened by EMA and National Competent Authorities (NCA) to support investigators in protocol development, with special focus on academic and SMEs-initiated trials, rare diseases trials, and trials on drug repurposing. EMA charges a fee for scientific advice, which varies depending on the scope of the advice.

Reductions apply for certain types of medicines and applicants, including a 75% fee reduction for medicines for orphan medicines and a 90% fee reduction for SMEs. Since 2020, academia is eligible for EMA protocol assistance (as a special form of scientific advice) free of charge for the development of orphan medicines. Some national competent authorities in Europe (for instance AEMPs, Spain) support informal consultations free of charge for investigator-initiated trials. More recently, in October 2021, a coordinated pilot program between EMA and HMA (Heads of Medicines Agencies) has been launched to support the repurposing of medicines [24]. The aim of this initiative is to support not-for-profit organizations and academia to gather or generate sufficient evidence on the use of an established medicine in a new indication with the view to have this new use formally authorized by a regulatory authority.

#### Funding and funding mechanism

While this may be a less pertinent problem for industry-funded trials, funding has been identified as a main challenge for all trials (Table 3). In the rare diseases area, the small patient population can dampen commercial interest, making this challenge more relevant for non-commercial RTC for rare diseases.

Five out of the six studies analyzed in this work were mainly funded through the E-Rare-3 JTC2016, based on a multinational funding scheme by national funding agencies (a “virtual common pot” mechanism, whereby each national funding body funds the national component of the trial, with no cross-border funding), while the DevelopAKUre program were granted under a central EU funding mechanism.

Some trials were funded through additional sources. HCQ4surfdefect trial was supported by three different funding schemes, in part consecutively, in part in parallel in full mutual recognition of the funders. For ROCK-ALS, investigators collected more than 100.00 EUR of donations to reimburse participants for travel to and from the trial site as well as for hotel costs. TAM DMD was supported also for two patients’ associations and the DevelopAKUre program got both European (FP7) and national (NHS) funding.

Restrictions on the use of national funds for crosscutting services and tendering rules were identified as important hurdles. Investigators funded through the multinational funding scheme agreed on the need of a funding scheme that allows for a mechanism (a real common pot, or a co-funding from the EU central budget, or any other funding source not restricted to a single country) to account for crosscutting activities and to allow flexibility in the budget allocation, reducing the administrative constraints of working with country-specific budgets. This administrative burden, the lack of flexibility to move transnationally funds, upper limits in the amount, and the duration of the funding hinder trial management, which often requires fast and frequent adaptation to changes in the recruitment rates, changes on exchange rates, different value added taxes (VATs), and other reasons out beyond control of investigators. This will have to be taken into consideration in the design of the ERA4HEALTH partnership, where a pillar will be dedicated to the funding (with an EU cofund) of multinational investigator-initiated clinical studies. Even though some sponsors underestimate the budget, in reality, many cost items may be impossible to foresee in advance. Poor awareness of Good Clinical Practice (GCP) requirements may lead sponsors to request insufficient financial resources from the funding agency, but in other cases, the budget awarded by the funding agency is much lower than the initially requested one and may be insufficient to meet all the cost required for full GCP compliance, i.e., adequate external monitoring or data management set up. Another cause for underestimation of the budget by a sponsor may also be the perception that a lower budget would decrease the likelihood of rejection. “Inadequate funding” was already identified as a barrier by several systematic reviews, but these sources referred mainly to the total cost estimation and did not specifically point out the lack of flexibility of the “funding mechanism” linked to public funding as a key obstacle.

#### Sponsorship

For non-industry sponsored trials, the sponsor is usually the institution to which the investigator is affiliated, a research foundation, and/or patient’s organization. Some of these academic/public institutions have previous experience and specific personnel to work as Sponsor’s representatives, but in other cases, the coordinating investigator assumes the sponsor’s role.

Three out of the six studies reported challenges linked to sponsor’s responsibilities: insufficient legal support from academic institutions acting as sponsors (Redox), no possible co-sponsorship (ROCK-ALS), and insufficient experience in quality assurance requirements (HCQ4SurfDefect) were identified as major hurdles.

For academic sponsored trials, generally one agency (or more in the case of the E-Rare funding scheme, which supported five of the six analyzed studies) provides the funding and another organization (usually the one that hosts the coordinating investigator) designs and conduct the trial in collaboration with research partners. The set-up of a complex multi-institutional partnership may sometimes be at odds with the concept of “single sponsor” which was developed for ensuring the protection of participants in the context of commercial trials. Indeed, single sponsorship is rooted in the need to clearly identify the legal responsibility and does not hinge upon the funding aspect. The ambiguity between the terms “funder” and “sponsor” [25] might contribute to the poor awareness of some inexperienced sponsors, especially in the non-commercial sector, of the scope of their own responsibilities. The legal sponsor is ultimately responsible for the scientific, ethical, regulatory, and legal aspects of the trial and also for the financial aspects (i.e., if an external funder withdraws, the sponsor will be responsible to look for funds to complete the trial). It is therefore its primary role and responsibility to ensure that sufficient resources are planned for full compliance with ethical and GCP requirements.

Interestingly, none of the previous systematic reviews had identified aspects linked to sponsorship as a main challenge, probably due to the fact that these reviews did not make a distinction between academic and industry sponsored trials. According to the provisions of the 536/2014 Clinical Trial Regulation, co-sponsorship should be an option to facilitate the sharing of tasks and responsibilities in multinational investigator-initiated trials.

#### Drug procurement

Drug procurement is generally perceived as a hurdle (four out of six investigators reported it as a challenge) due to (a) the lack of harmonization among countries on the requirements for Quality assurance, i.e., stability of the drug after re-conditioning, re-formulation, (b) need for a tendering process, and (c) the high cost of drug distribution. The ROPROP and the HCQ4SurDef studies were both requested to present additional stability data for repurposed drugs only in some countries. Redox’s trial coordinator perceived the tendering process as especially cumbersome, and the ROCK-ALS coordinator described as a special challenge the procedure to import the drug from a non-European country.

A recent meta-research study [26] describes a series of challenges in obtaining placebo for investigator-initiated drug trials, including the high cost for production and packaging and the unwillingness of manufactures to provide placebos for RCTs without previous assessment and approval of protocols [27]. Five of the six trials analyzed in this work were placebo-controlled. Two of them received placebo from the drug manufacturer, while for the other three, a hospital pharmacy was involved in the manufacturing and distribution. One investigator reported unwillingness/unavailability of the manufacturer to provide the study drug.

#### Project management

As reflected by the many aspects linked to the lack of harmonization among countries described in this and previous works, management of multinational trials requires experienced academic clinical trial units (CTUs). In many cases, CTUs, publicly funded at national level to support in-house investigators, have limited experience supporting multi-country studies. Indeed, differential globalization of industry and non-industry sponsored trials has been reported [5] as academic-sponsored trials are mainly conducted in a single country.

Investigators relied in both local academic clinical trial units/research units (four out of six studies) and CROs (two out of six studies) for global project management. Only one coordinating investigator reported project management as a challenge linked to the limited previous experience of the CTU on multinational management. Fortunately, the reported increase in the number of academic-sponsored trials over the last years [5] should lead to an improvement on the investment to promote the important role of academic clinical trial units and international research infrastructures (ref www.ecrin.org) on international clinical trials management.

#### Data management

Managed by both CTUs and CROS for the analyzed RCTs, only one of the trial coordinators reported data management as a main hurdle. This might reflect sponsor’s awareness on the need to maintain a good quality control system, as data management was identified as one of the top critical GCP findings [28] during EMA inspections. Most of the critical findings are related to insufficient quality control (e.g., edit checks) performed on the data captured in the database considering that relevant inconsistencies in the data were not recognized and not followed up.

#### Vigilance and monitoring

Pharmacovigilance and monitoring was generally not perceived as a hurdle. Only one trial’s coordinator described the lack of experience of the initially selected CTU to perform pharmacovigilance at multinational level.

### Country set up

#### Country selection

Country selection was not perceived as a hurdle as such, but some investigators pointed out that the funding scheme might favor the selection of participating countries based on the funding rules (specially for co-fund programs) instead of being driven by a feasibility analysis of adequate country sites, investigators, and patients.

#### NCA/EC approvals

Timing and lack of harmonization is generally perceived as a major hurdle. The Voluntary Harmonization Procedure (VHP) was conceived to harmonize and coordinate procedures and to streamline the review process of competent authority applications for multi-center/multinational clinical trials, while the implementation of the 2014 CTR (Clinical Trials Regulation) [29] was delayed. However, there is no legal obligation for the EU countries to take part in this process. Investigators were interrogated about the use of VHP for regulatory submission. Interestingly, none of the studies applied for approval through VHP. Moreover, two studies reported having withdrawn VHP as it was perceived as more time consuming and less flexible that single country-per-country applications even though the procedure has received increasing acceptance since its introduction in 2009 [30]. Published sponsor’s experience on the use of VHP is scarce, and while the first commercial sponsor to use VHP reported a positive experience [31], others did not report special benefit over the use of multiple national applications [32]

#### Insurance coverage

Insurance or indemnity to cover the liability of the investigator and sponsor must be secured before the study commences. The “obligatory insurance” was introduced in Europe with the implementation of Directive 2001/20/EC. This insurance was meant to cover the sponsor in case of liability for injury or death to participants and the terms depend on the individual risk and number of patients. In the USA, institutions are usually covered by their institution liability insurance. Cost and discordant requirements for insurance and indemnification in Europe has been previously reported to challenge the RCT set up at country level [15, 16] leading to recommendations to better inform investigators and sponsors about the rational for the requirements in each country [33]. Interestingly, with the 2014 Clinical Trials Regulation [29], national indemnification is not applicable to low-intervention clinical trials. While this might favor especially implementation of academic-sponsored RTC on drug repurposing, the definition of “low-intervention trial” still requires a determination as to which trials pose “minimal additional risk or burden.” For now, the term seems open to interpretation, which might hinder the financial benefit of “insurance exemption”.

Insurance handling was nevertheless not perceived as a hurdle. E-Rare-3 funded studies planned insurance handling as a national coordinator’s responsibility, facilitating country-specific contracting and preventing the sponsor from having to deal with not-harmonized rules in terms of coverage requirements.

#### Good clinical practice (GCP) training

One study highlighted lack of harmonization on requirements/content of GCP training. While European Clinical Trials legislation requires all clinical trials being conducted in accordance with the principles of GCP, personnel GCP training is required to get the study approved and/or initiated in all countries. Nevertheless, the content and extent of GCP training, the site personnel requiring the training, and the requirements to obtain GCP certificates are not harmonized, varying from country to country [34]. Indeed, while most commercial sponsors have developed sponsor-specific GCP training based on industry-agreed standards (Transcelerate mutual recognition) [35], ethics committees might require and/or recommend for both commercial and academic trials, specific training adapted to local specificities and in local language.

### Site set up

#### Site agreements

Generally perceived as a hurdle linked to multiple aspects as:

- Lack of harmonization on site agreement templates, even within the same country
- Country-specific terminology and interpretation of legal responsibilities, intellectual/industrial property, GDPR (General Data Protection Regulation)
- Reference to the national legal and regulatory framework (liability and insurance)
- Cost variability (applicable taxes/overheads)
- Translations
- Templates tailored to industry-sponsored trials, not aligned with Consortium and Grant Agreements linked to publicly funded studies

Site agreement management challenges especially academic sponsor’s legal departments due to the lack of experienced personnel and/or limited resources.

Four out the six trials highlighted site contracting as a main operational constraint. Local site agreements often create the greatest time delay, and pragmatic, sensible legal, and administrative solutions are required. In this sense, the development of a pan-European site agreement template has been proposed [32]. Acceptance of this template by implementing site/institutions could be an eligibility criterion for publicly funded/multinational trials in the EU.

#### Site compliance

Many academic researchers at academic institutions struggle with short-term employment contracts and the pressure to publish rapidly. Engagement in long-term strategic clinical research might be considered as counterproductive for the individual clinician because results might remain unpublishable for many years. Lack of personnel to work in academic-sponsored trials was not considered a hurdle for the analyzed use cases.

## Conclusions

Multinational clinical trials for drug repurposing in rare diseases are mostly initiated by academia [6], but sponsors and investigators leading these trials face a wide range of challenges than hinder patient’s access to drugs. Main hurdles are more operational than scientific, ranging from regulatory submission to site contracting. Sponsorship requirements as well as “funding mechanism” have not been pointed out before as an important challenge for academia. Limited funding had been previously described as an obstacle but it is actually the public funding mechanism, without cross-border budget flexibility, more than the total amount, what is perceived as an important hurdle. There are no main clinical trials’ academic-sponsor advocacy groups in Europe and academy have very little presence in clinical trials’ European forums, which has led to the development of regulations and guidelines that might suit better pharmaceutical industry’s needs. A better knowledge of the non-commercial clinical research landscape and its challenges and requirements is needed to achieve RD academic clinical research’s main goal, which is making drugs—especially those with less commercial gain—accessible to rare diseases patients. Better information about existing resources like research infrastructures, clinical research programs, and counseling mechanisms is needed to support and guide clinicians through the many challenges associated to the set-up of academic-sponsored multinational trials.

## Data Availability

Not applicable.

## Abbreviations

AEMPS: Agencia Española de Medicamentos y Productos Sanitarios (Spanish drug and medical devices agency)
CRO: Clinical Research Organization
CTU: Clinical trial unit
EC: Ethics Committee
EJP: European Joint Programme on Rare Diseases
EMA: European Medicines Agency
EU: European Union
FP7: 7th Framework Program
GCP: Good Clinical Practice
GDPR: General Data Protection Regulation
HMA: Heads of Medicines Agencies
NCA: National Competent Authority
NCT: National clinical trial
NHS: National Health Service
RCT: Randomized clinical trial
SME: Small and medium-sized enterprises
VHP: Voluntary Harmonization Procedure
